# Percutaneous Mechanical Thrombectomy of a Superior Vena Cava and Right Atrial Thrombus Complicating a Malfunctioning Tunneled Dialysis Catheter

**DOI:** 10.7759/cureus.115590

**Published:** 2026-09-01

**Authors:** Renato Abu Hana, Grit A Adler, Ryosuke Miyauchi, Vinicius Adami Vayego Fornazari

**Affiliations:** 1 Radiology, University of Florida College of Medicine – Jacksonville, Jacksonville, USA

**Keywords:** central venous stenosis, clottriever, hemodialysis, interventional radiology, mechanical thrombectomy, right atrial thrombus, superior vena cava, tunneled dialysis catheter

## Abstract

Tunneled dialysis catheters are a mainstay of hemodialysis access but are complicated over time by central venous stenosis and catheter-related thrombus, both of which can impair catheter function and threaten future access. We describe the case of a 77-year-old woman who required hemodialysis for acute kidney injury on underlying chronic kidney disease and, within weeks of placement of her first right internal jugular tunneled dialysis catheter, developed superior vena cava (SVC) stenosis and an organized thrombus at the cavoatrial junction extending into the right atrium (RA). The thrombus caused catheter malfunction with low flow and clotting. Cardiac computed tomography confirmed the SVC/RA thrombus. In a single percutaneous session, mechanical thrombectomy, SVC angioplasty, and catheter exchange achieved complete thrombus clearance and a widely patent SVC, and restored catheter function without thrombolysis or open surgery. This case illustrates thrombolytic-free mechanical thrombectomy as an effective, thrombolytic-free treatment option for catheter-associated SVC/RA thrombus that preserves dialysis access in a single sitting.

## Introduction

Tunneled dialysis catheters (TDCs) provide reliable central venous access for patients with end-stage renal disease, but prolonged catheter dwell time carries a recognized burden of complications. These span infectious complications, chiefly catheter-related bloodstream infection; thrombotic and occlusive complications, including fibrin sheath formation, intraluminal and mural thrombus, and central venous occlusion; and stenotic complications, principally central venous stenosis (CVS). Chronic contact between the catheter and the vessel wall produces endothelial injury, intimal hyperplasia, and fibrosis that underlie CVS, while mechanical irritation of the right atrium (RA) and disturbed local flow promote catheter-related right atrial thrombus (CRAT) [1,2]. These processes converge on the same clinical endpoint: catheter dysfunction, rising venous pressures, incomplete dialysis, and, in advanced cases, superior vena cava (SVC) obstruction and loss of the access site [3]. Although these complications are classically associated with long-term catheter dwell, they can develop within weeks of catheter placement, as in the present case.

CRAT in hemodialysis patients is uncommon but potentially fatal, with reported incidences ranging widely and mortality historically approaching one in five [3,4]. Optimal treatment remains unsettled. Reported strategies include catheter removal or exchange, systemic anticoagulation, thrombolysis, open surgical thrombectomy, and percutaneous mechanical thrombectomy, each with distinct limitations in a population marked by bleeding risk, cardiovascular comorbidity, and dependence on the affected access [3-5]. Anticoagulation is often preferred but acts slowly and carries hemorrhagic risk; thrombolysis adds further bleeding hazard; and surgical thrombectomy requires sternotomy and cardiopulmonary bypass in patients who are frequently poor operative candidates [5-7]. Thrombolytic-free mechanical thrombectomy has been used to remove thrombus in a single session while avoiding lytic agents; however, the supporting evidence derives largely from peripheral deep venous thrombosis [8], and its application to catheter-associated SVC and RA thrombus is currently limited to case reports and small series [9].

We report the endovascular management of an organized SVC and RA thrombus that arose within weeks of a first TDC and coexisted with SVC stenosis, presenting with catheter malfunction. Mechanical thrombectomy, SVC angioplasty, and catheter exchange were combined in one session to clear the thrombus and preserve dialysis access. In the present case, several factors favored immediate mechanical thrombectomy over initial anticoagulation alone: a large, organized-appearing thrombus spanning the cavoatrial junction, recurrent catheter dysfunction, dependence on the catheter for dialysis access, and the need for prompt restoration of catheter function.

## Case presentation

A 77-year-old woman with type 2 diabetes mellitus, hypertension, chronic kidney disease, heart failure with preserved ejection fraction, peripheral vascular disease, chronic obstructive pulmonary disease on home oxygen, obstructive sleep apnea, and prior cerebrovascular accident was hospitalized for a non-ST-elevation myocardial infarction and developed severe anuric acute kidney injury on underlying chronic kidney disease, necessitating initiation of hemodialysis through her first right internal jugular (IJ) TDC. Within weeks of placement, the catheter developed progressive dysfunction, with low blood flow and recurrent clotting during dialysis. The patient had no symptoms of SVC syndrome such as facial or upper-extremity swelling. Laboratory values at dialysis initiation and around the procedure are summarized in Table 1.

**Table 1:** Laboratory values at dialysis initiation, on the day of thrombectomy, and the following day. Laboratory values at dialysis initiation (April 2026), on the day of percutaneous thrombectomy (five weeks later), and the following day after the dialysis session. Reference ranges are shown in the final column. Hemoglobin remained stable across the procedure, consistent with minimal procedural blood loss.

Laboratory test	Dialysis initiation	Procedure day (5 weeks later)	Post-dialysis (1 day after )	Reference range
Creatinine (mg/dL)	5.65	5.04	3.28	0.6–1.1
Blood urea nitrogen (mg/dL)	10.7	5.8	7.8	7–20
Potassium (mEq/L)	5.5	5.1	4	3.5–5.1
Hemoglobin (g/dL)	9.2	8.3	8.4	12.0–15.5
Hematocrit (%)	30	28.2	28.7	36–46
Platelet count (×10³/µL)	258	258	258	150–400
International normalized ratio	1	1.1	1	0.8–1.1

Contrast-enhanced cardiac computed tomography (CT) demonstrated an organized thrombus (approximately 9 × 10 × 43 mm) involving the lower SVC and extending across the cavoatrial junction into the RA, accounting for the recurrent catheter malfunction (Figure 1). A therapeutic heparin infusion (80 units/kg bolus, followed by a weight-based infusion titrated to a therapeutic activated partial thromboplastin time) was initiated upon identification of the thrombus. Given the thrombus location and extension, percutaneous mechanical thrombectomy was planned.

**Figure 1:**
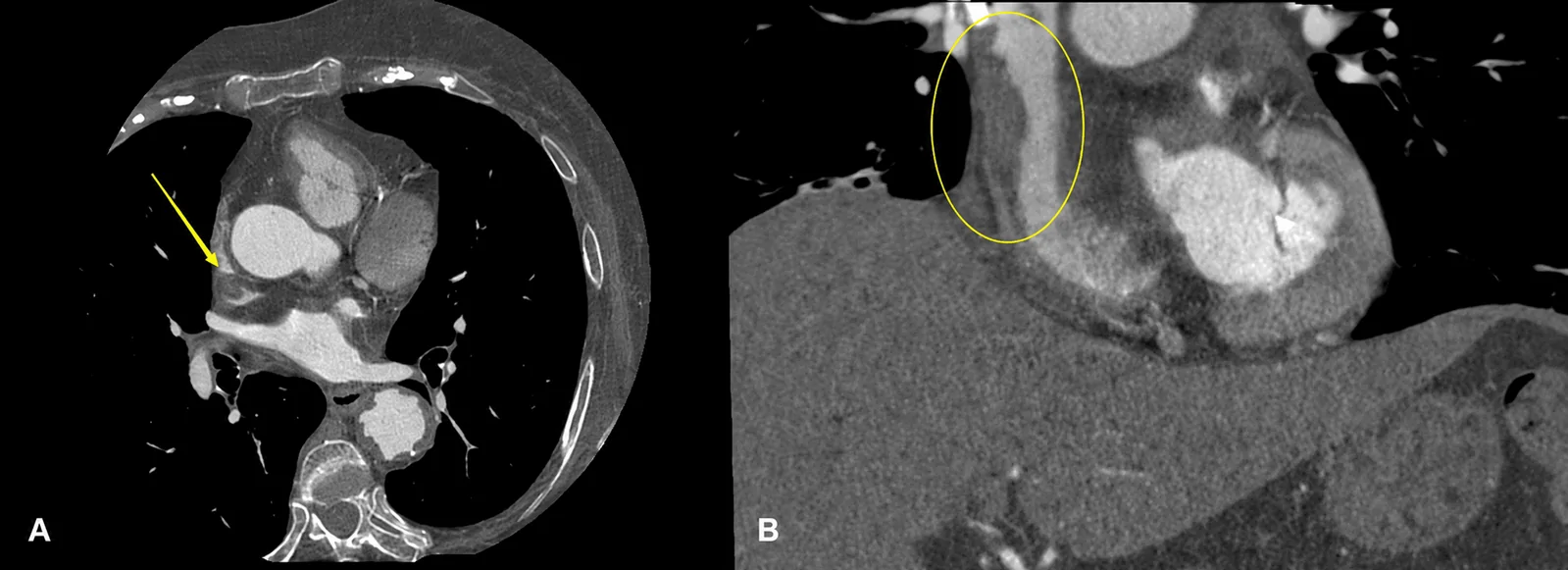
Cardiac computed tomography. Contrast-enhanced cardiac computed tomography of the superior vena cava (SVC) and right atrial thrombus. (A) Axial image demonstrating a hypoattenuating intraluminal filling defect within the SVC (arrow), consistent with organized thrombus. (B) Coronal reformatted image showing the filling defect (circle) extending across the cavoatrial junction into the right atrium (RA); the hyperattenuating focus corresponds to the indwelling catheter. The thrombus measured approximately 9 × 10 × 43 mm (transverse × anteroposterior × craniocaudal).

The procedure was performed under moderate sedation. The right neck and chest were prepared and draped in sterile fashion. Given the potential for clot fragmentation and pulmonary embolism during manipulation of the organized thrombus, the patient was continuously monitored, with vascular access and the thrombectomy device maintained in position to permit immediate intervention in case of an acute pulmonary embolism; no such event was observed.

The in-place TDC was retracted to the brachiocephalic vein, and superior vena cavography performed through the catheter demonstrated organized thrombus adherent to the lower SVC and RA (Figure 2). Under real-time ultrasound guidance, the right IJ vein was then accessed with a 21-gauge needle, and a guidewire was advanced into the inferior vena cava (IVC). Following serial dilation, a 16-French Inari ClotTriever sheath (Inari Medical, Irvine, California, USA) was placed.

**Figure 2:**
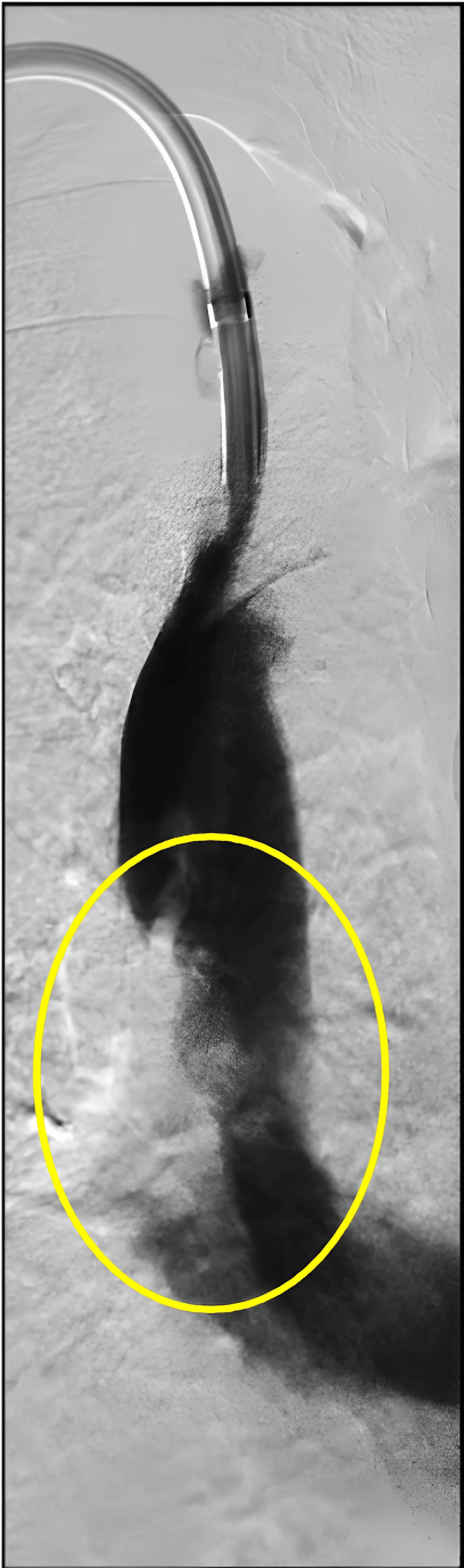
Initial superior vena cavogram demonstrating the superior vena cava and right atrial thrombus. Digital subtraction venogram obtained through the right internal jugular access. The yellow ellipse outlines a large intraluminal filling defect adherent to the lower superior vena cava, extending across the cavoatrial junction toward the right atrium, consistent with organized thrombus.

To preserve dialysis access, a stiff Glidewire was advanced through the in-place TDC into the IVC, and the catheter was tracked over the wire. The ClotTriever XL system was then introduced through the right IJ sheath, and the collection element was deployed distal to the thrombus (Figure 3A). Several coring passes achieved substantial clot removal (Figure 4). Post-thrombectomy venography showed near-complete resolution of the thrombus. The SVC was subsequently treated with angioplasty using 16- and 18-mm Atlas balloons (BD, Tempe, Arizona, USA) (Figure 3B).

**Figure 3:**
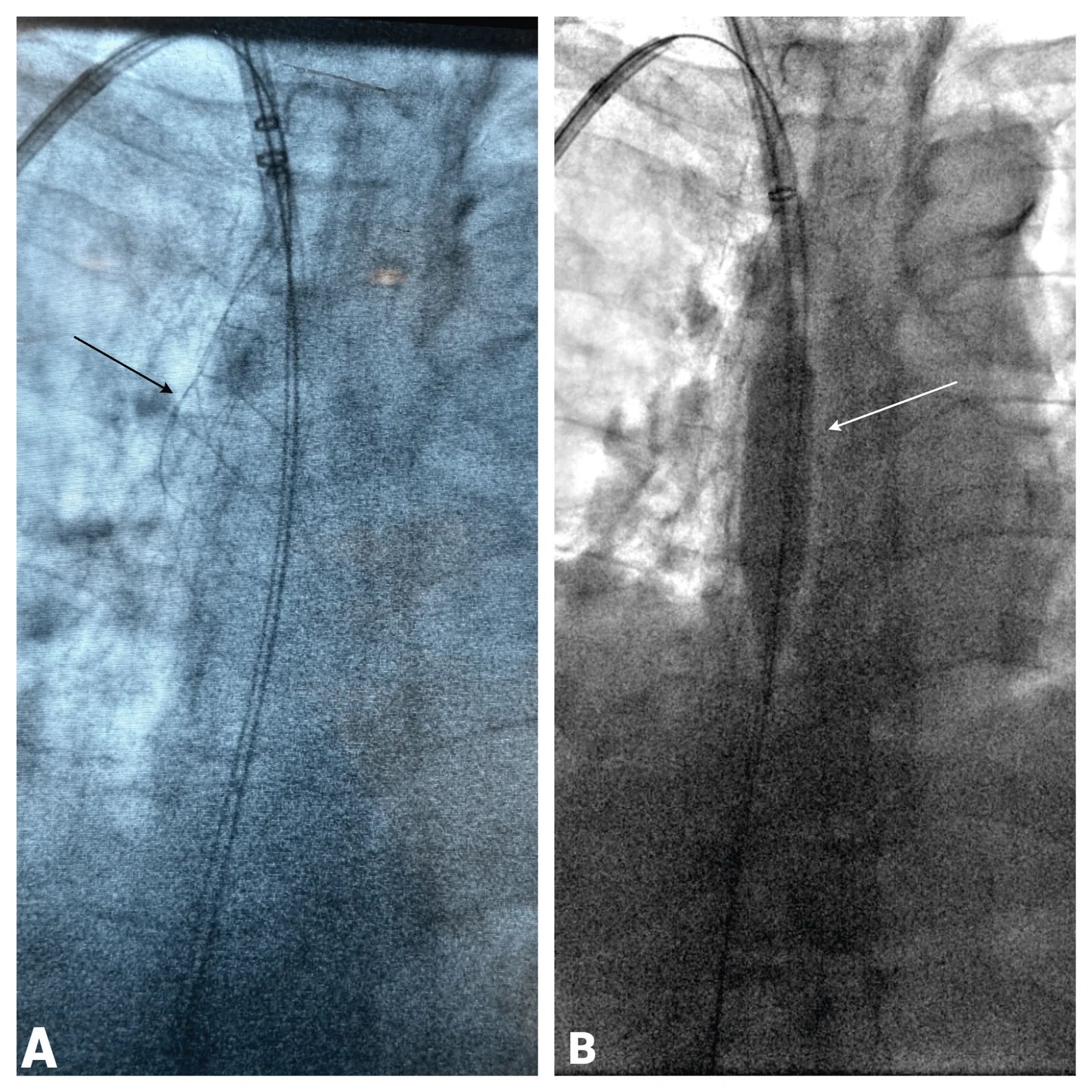
Intraprocedural fluoroscopy during mechanical thrombectomy and superior vena cava angioplasty. Intraprocedural fluoroscopy during mechanical thrombectomy and superior vena cava angioplasty. (A) The Inari ClotTriever collection element (clot basket) deployed within the superior vena cava during mechanical thrombectomy (black arrow). (B) Angioplasty of the superior vena cava; the 18-mm Atlas balloon (BD, Tempe, Arizona, USA) is shown inflated across the stenosis (white arrow). The stenosis was treated sequentially with 16- and 18-mm balloons.

**Figure 4:**
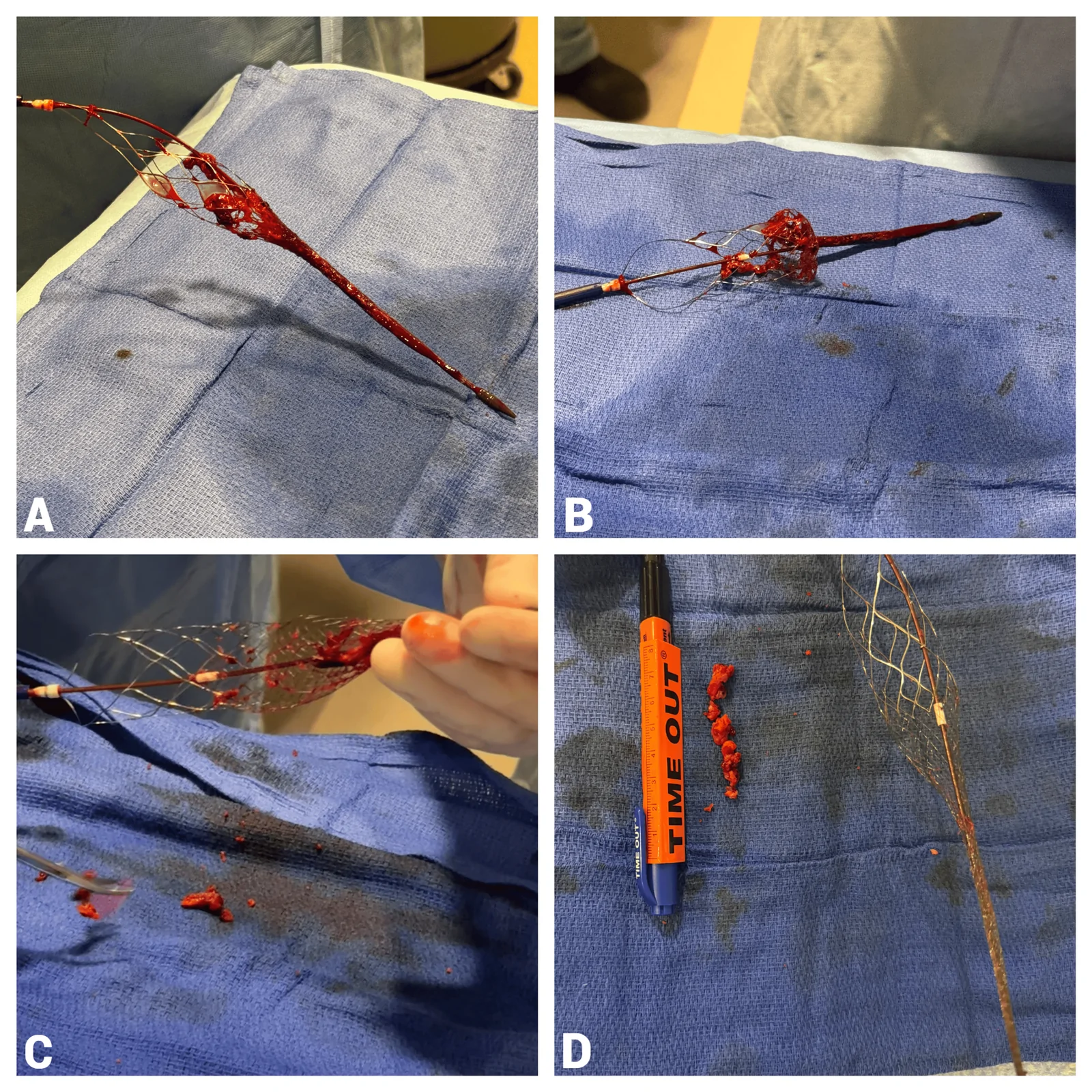
Mechanically extracted organized thrombus. (A, B) The ClotTriever retrieval device with its nitinol collection element and adherent organized thrombus removed from the superior vena cava and right atrium. (C) Close-up of the collection element containing the retrieved thrombus. (D) Extracted thrombus fragments alongside a pen for scale.

Final venography confirmed complete clearance of the thrombus with a widely patent SVC (Figure 5A). A new 14.5-French, 19-cm high-flow tunneled hemodialysis catheter (Palindrome Chronic Dialysis Catheter; Medtronic, Minneapolis, Minnesota, USA) was advanced over the preserved guidewire, with its tip positioned at the cavoatrial junction. Fluoroscopy confirmed the absence of kinking, and test aspiration demonstrated brisk flow through both lumens (Figure 5B). The catheter was secured to the chest wall with a Prolene suture, and both ports were locked with sodium citrate solution per protocol. A purse-string suture was placed at the right neck access site to permit sheath removal, and hemostasis was achieved. The patient tolerated the procedure without immediate complications.

**Figure 5:**
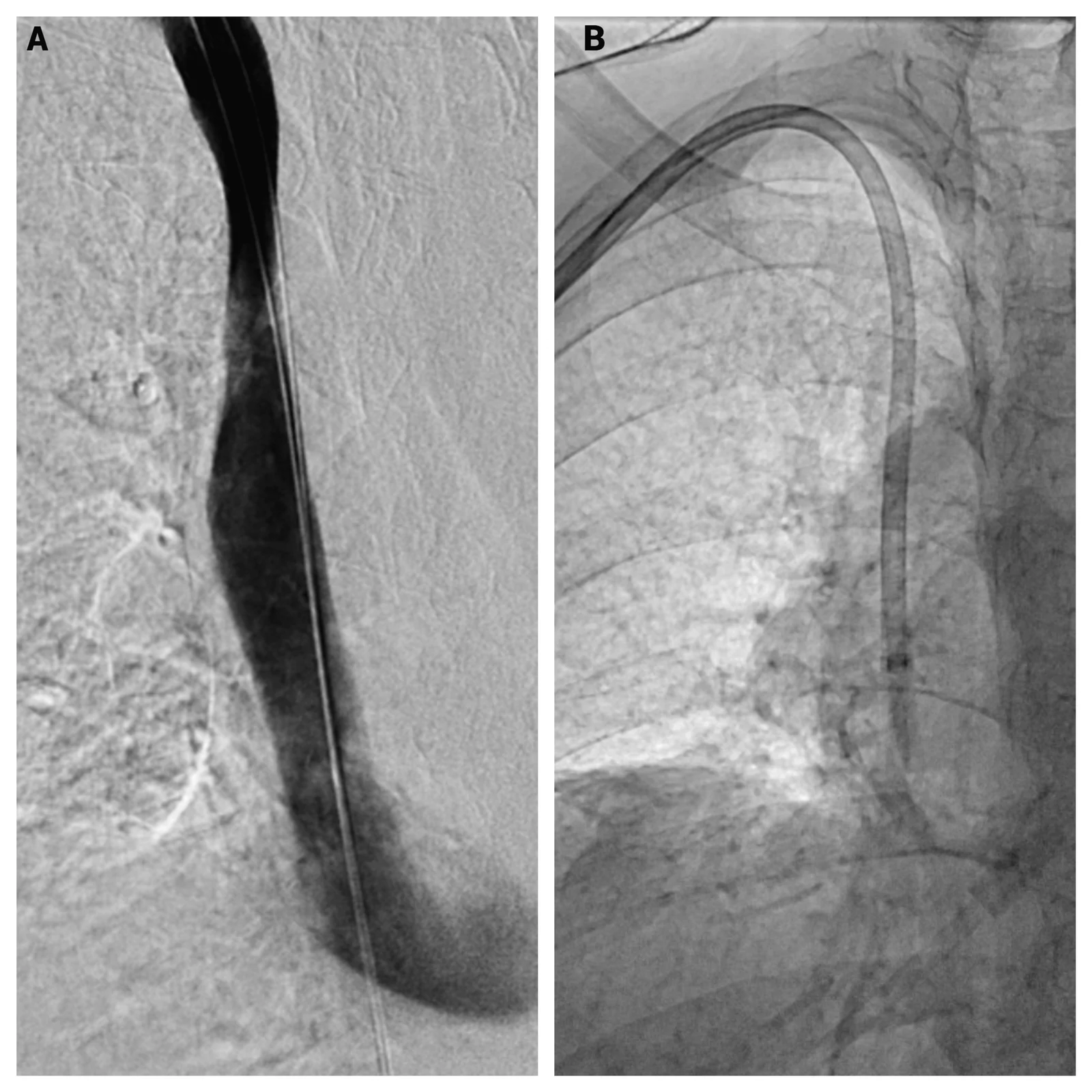
Post-procedure imaging. (A) Superior vena cava (SVC) venogram following mechanical thrombectomy and angioplasty showing complete resolution of the previously demonstrated adherent thrombus at the SVC/right atrial junction. (B) Post-procedure fluoroscopy showing the new tunneled dialysis catheter in appropriate position.

Immediately following the procedure, the patient underwent hemodialysis and completed a full three-hour session with satisfactory blood flow and no complications. Unfractionated heparin was continued peri-procedurally, and the patient was subsequently transitioned to warfarin 5 mg orally daily (target international normalized ratio: 2.0-3.0), planned for a minimum of three months and while the TDC remained in place, to mitigate residual thrombogenic risk after mechanical thrombus removal. No bleeding complications occurred. At the time of this report, she continued thrice-weekly hemodialysis through the catheter without recurrence of dysfunction.

## Discussion

This case brings together two related complications of TDC use, namely, CVS and CRAT, and shows that both can be addressed in a single percutaneous session. The shared origin of these entities is instructive. Indwelling catheters injure the venous endothelium and RA endocardium through direct mechanical contact and pulsatile motion of the catheter tip, triggering intimal hyperplasia and fibrosis that narrow the SVC and exposing a thrombogenic surface on which clot organizes [1,2]. Catheter tip position within the RA is itself correlated with atrial thrombus formation, and the resulting thrombus tends to be organized and adherent rather than fresh and mobile [2,3]. Although CVS and CRAT are more commonly associated with long-dwelling catheters, this case demonstrates that both can arise within weeks of a first catheter; an early presentation should therefore not lower clinical suspicion for these complications. In this patient, the thrombus straddled the cavoatrial junction and coexisted with SVC stenosis, a combination that catheter exchange or balloon angioplasty alone would not resolve, as neither maneuver removes an organized junctional clot.

The management of catheter-associated RA thrombus is not standardized, and the available options each carry meaningful drawbacks in a hemodialysis population. Systematic reviews of reported cases favor catheter removal or exchange combined with anticoagulation, reserving thrombectomy for failure of medical therapy or for new complications [3,4]. Anticoagulation, however, dissolves organized thrombus slowly and unreliably and exposes uremic patients to bleeding; thrombolysis compounds that hazard and is frequently contraindicated; and open surgical embolectomy demands cardiopulmonary bypass in patients who often tolerate it poorly [3-7]. Reported experience with these approaches includes both surgical embolectomy for large adherent thrombi [7] and, more recently, thrombolytic-free mechanical thrombectomy of catheter-related atrial thrombus using the Inari platform [8]. The present case adds to that experience and extends it to a thrombus that had organized across the SVC and RA in the setting of fixed venous stenosis.

In a systematic review of catheter-related right atrial thrombosis, the median time to diagnosis in hemodialysis patients was 12 weeks (interquartile range: 4-24 weeks), indicating that although most cases present later, a substantial proportion arise within the first weeks after catheter placement [9].

Mechanical thrombectomy was selected here not as a last resort but as the treatment best matched to the problem. A large organized clot at the cavoatrial junction is unlikely to resolve with anticoagulation within a clinically acceptable window, and the patient’s dependence on the access made prompt restoration of catheter function a priority. The ClotTriever system removes thrombus by advancing a nitinol coring element and collection element through the clot and drawing the material into a bag, without thrombolytics. In the 500-patient CLOUT registry, it achieved complete or near-complete thrombus removal in the great majority of limbs, predominantly in a single session and with low blood loss, across a range of clot chronicity; however, these data derive from lower-extremity deep venous thrombosis and are only indirectly applicable to an organized cavoatrial thrombus [10]. Treating the underlying SVC stenosis with angioplasty in the same sitting addressed the fixed lesion that had contributed to recurrent catheter failure, and exchanging the catheter over a preserved wire re-established a functioning access without a separate procedure or a new venipuncture through diseased vein.

Several features of the technique merit emphasis. Access was obtained with the existing catheter maintained on a safety wire, so dialysis access was never lost during the intervention and could be exchanged for a new catheter at the conclusion of the case. Working within the RA and at the cavoatrial junction carries a recognized, if small, risk of atrial wall injury, pericardial effusion, arrhythmia, and clot fragmentation with pulmonary embolization. Dislodgement of an organized junctional thrombus into the pulmonary circulation is a particular concern during clot manipulation. In this case, that risk was mitigated by continuous hemodynamic and respiratory monitoring throughout the procedure and by maintaining vascular access and the thrombectomy device in position, so that an acute pulmonary embolism could have been addressed immediately with aspiration or repeat thrombectomy. Limiting the number of coring passes reflected the operators’ rationale for reducing embolic risk rather than an established risk-reduction strategy. Avoiding thrombolysis is particularly advantageous in patients with renal failure, in whom platelet dysfunction and comorbidity magnify bleeding risk.

The limitations of a single case apply. This report describes technical and immediate clinical success; durable patency of the SVC and the exchanged catheter, the optimal anticoagulation strategy after thrombectomy, and the risk of recurrent stenosis or thrombus can only be established with longer follow-up and larger series.

## Conclusions

TDCs are indispensable for hemodialysis but carry a spectrum of complications that can compromise catheter function and jeopardize future access, including CVS, fibrin sheath formation, thrombosis, occlusion, and infection. Catheter-associated SVC and RA thrombus is among the most serious of these, and its management remains without consensus. The available options, including anticoagulation, thrombolysis, surgical thrombectomy, and percutaneous mechanical thrombectomy, are each constrained by bleeding risk, comorbidity, or operative morbidity in this population. This case demonstrates the technical feasibility of thrombolytic-free percutaneous mechanical thrombectomy combined with SVC angioplasty and catheter exchange for a large catheter-associated SVC/RA thrombus in a patient requiring ongoing dialysis access. The approach achieved immediate thrombus clearance and restoration of catheter function without an observed procedural complication. Larger studies and longer follow-up are required to define patient selection, procedural safety, anticoagulation strategy, recurrence risk, and long-term patency.
